# 3D Bioprinted Bacteriostatic Hyperelastic Bone Scaffold for Damage-Specific Bone Regeneration

**DOI:** 10.3390/polym13071099

**Published:** 2021-03-30

**Authors:** Mohammadreza Shokouhimehr, Andrea S. Theus, Archana Kamalakar, Liqun Ning, Cong Cao, Martin L. Tomov, Jarred M. Kaiser, Steven Goudy, Nick J. Willett, Ho Won Jang, Christopher N. LaRock, Philip Hanna, Aron Lechtig, Mohamed Yousef, Janaina Da Silva Martins, Ara Nazarian, Mitchel B. Harris, Morteza Mahmoudi, Vahid Serpooshan

**Affiliations:** 1 Department of Materials Science and Engineering, Research Institute of Advanced Materials, Seoul National University, Seoul 08826, Korea; shokouhimehrm@gmail.com (M.S.); hwjang@snu.ac.kr (H.W.J.); 2 Department of Biomedical Engineering, Georgia Institute of Technology, School of Medicine, Emory University, Atlanta, GA 30322, USA; andrea.theus@emory.edu (A.S.T.); liqun.ning@emory.edu (L.N.); martin.lyubomirov.tomov@emory.edu (M.L.T.); nick.willett@emory.edu (N.J.W.); 3 Department of Otolaryngology, School of Medicine, Emory University, Atlanta, GA 30322, USA; archana.kamalakar@emory.edu (A.K.); Steven.goudy@emory.edu (S.G.); 4 Department of Physics, Emory University, Atlanta, GA 30322, USA; cong.cao@emory.edu; 5 Department of Orthopedics, Emory University, Atlanta, GA 30322, USA; jarred.kaiser@emory.edu; 6 Atlanta Veteran’s Affairs Medical Center, Decatur, GA 30033, USA; 7 Department of Microbiology and Immunology, School of Medicine, Emory University, Atlanta, GA 30322, USA; christopher.larock@emory.edu; 8 Center for Advanced Orthopaedic Studies, Beth Israel Deaconess Medical Center, Harvard Medical School, Boston, MA 02215, USA; philiphanna2020@gmail.com (P.H.); alechtig@bidmc.harvard.edu (A.L.); anazaria@bidmc.harvard.edu (A.N.); 9 Department of Orthopedic Surgery, Sohag University, Sohag 82524, Egypt; dr_mohamed_222@yahoo.com; 10 Endocrine Unit, Massachusetts General Hospital, Harvard Medical School, 50 Blossom St, Thier 11, Boston, MA 02114, USA; JDASILVAMARTINS@mgh.harvard.edu; 11 Department of Orthopaedic Surgery, Yerevan State Medical University, Yerevan 0025, Armenia; 12 Orthopaedic Surgery, Massachusetts General Hospital, Harvard Medical School, Boston, MA 02115, USA; MBHARRIS@mgh.harvard.edu; 13 Precision Health Program & Department of Radiology, Michigan State University, East Lansing, MI 48824, USA; mahmou22@msu.edu; 14 Department of Pediatrics, School of Medicine, Emory University, Atlanta, GA 30322, USA; 15 Children’s Healthcare of Atlanta, Atlanta, GA 30322, USA

**Keywords:** damage-specific scaffold, bone 3D bioprinting, tissue engineering, hyperelastic bone, superparamagnetic iron oxide nanoparticles, antibacterial, large bone fracture

## Abstract

Current strategies for regeneration of large bone fractures yield limited clinical success mainly due to poor integration and healing. Multidisciplinary approaches in design and development of functional tissue engineered scaffolds are required to overcome these translational challenges. Here, a new generation of hyperelastic bone (HB) implants, loaded with superparamagnetic iron oxide nanoparticles (SPIONs), are 3D bioprinted and their regenerative effect on large non-healing bone fractures is studied. Scaffolds are bioprinted with the geometry that closely correspond to that of the bone defect, using an osteoconductive, highly elastic, surgically friendly bioink mainly composed of hydroxyapatite. Incorporation of SPIONs into HB bioink results in enhanced bacteriostatic properties of bone grafts while exhibiting no cytotoxicity. In vitro culture of mouse embryonic cells and human osteoblast-like cells remain viable and functional up to 14 days on printed HB scaffolds. Implantation of damage-specific bioprinted constructs into a rat model of femoral bone defect demonstrates significant regenerative effect over the 2-week time course. While no infection, immune rejection, or fibrotic encapsulation is observed, HB grafts show rapid integration with host tissue, ossification, and growth of new bone. These results suggest a great translational potential for 3D bioprinted HB scaffolds, laden with functional nanoparticles, for hard tissue engineering applications.

## 1. Introduction 

The quest to develop an ideal bone graft that is compatible with the extensive variety of osseous-tissue-related medical indications has been an ongoing challenge [1,2]. Although there are numerous clinical products used today as bone void fillers [3,4] or temporary scaffolds [5,6], there has been an extensive body of literature reporting the potential of new bone-related biomaterials [7,8]. These products and their surgical implementation suffer from substantial technical, surgical, and manufacturing/scaling shortcomings [2,9]. Successful applications of bone tissue engineering can replace traditional treatment options that involve materials such as autografts or allografts [10], which have limited supplies. Furthermore, these traditional treatment options are often associated with donor-site morbidity and have the possibility of immune rejection and transmission of diseases [11,12]. While tissue engineered scaffolds are becoming an integral part of developing the ideal bone graft, their clinical translation still faces several challenges on achieving effective osteointegration and vascularization [13]. Multidisciplinary approaches must be taken to ensure the accelerated processes of osteogenesis and osteointegration and to facilitate clinical applications [2,14]. 

Three-dimensional (3D) printing and bioprinting methods allow for precise, reproducible, and large-scale fabrication of complex scaffolding systems with tunable architecture and physiomechanical properties [15,16]. In addition, medical imaging data can be employed to bioprint implants with patient- and damage-specific geometries [17,18]. Bioprinting techniques are of particular interest in bone tissue engineering, where there is an extensive need for the development of grafts for critical size defects (when the length of the defect is 2–3 times its diameter) [19,20,21,22]. The manipulation of computer-aided design (CAD) models, used for printing scaffolds, allows for precise tuning of porosity, size, and geometric design of the graft to closely correspond with the target bone defect [23].

While a variety of bioink materials have been tested in additive manufacturing of bone-like scaffolds, demonstrating promising in vitro and in vivo osteoconductivity, such scaffold-based therapies have shown only a modest success in clinical applications [24,25]. This is mainly due to the lack of proper osteoconductive cues in the bioink, biomaterial-related infections, insufficient engraftment, and inadequate reproducibility and patient-specificity [26,27]. Hyperelastic bone (HB) is a promising osteoregenerative bioink that could be printed into complex geometries at high manufacturing rates (up to 275 cm^3^/hour) [28]. This biomaterial contains up to 90% hydroxyapatite and displays elastic properties due to the incorporated polylactic-co-glycolic acid (PLGA) polymer [28,29]. Without the need for added biological factors, HB bioink can support new bone formation and vascularization, owing to its unique biomechanical and biochemical properties [29,30,31].

In this study, we demonstrated the use of HB bioink to 3D bioprint reproducible, highly porous, and damage-specific bone grafts. Further, to confer bacteriostatic properties to the implants, superparamagnetic iron oxide nanoparticles (SPIONs) were incorporated into the HB bioink. We assessed the osteoregenerative capacity of bioprinted scaffolds both in vitro and in vivo, in a critical-sized bone defect in the rat femur.

## 2. Materials and Methods

### 2.1. 3D Bioprinting of Bone Constructs

3D printed bone constructs were prepared using the HB ink (Dimension Inx, Chicago, IL, USA) [28]. The HB paint is composed of 90% weight (wt) hydroxyapatite and 10% wt poly(lactic-co-glycolic acid). Prepared SPIONs were incorporated into the HB ink to yield concentrations of 60 µg/mL and 200 µg/mL of SPIONs (clinically available ferumoxytol; FERAHEME^®^ [32,33,34]. These concentrations were chosen based on the cytotoxicity threshold of 100 µg/mL for these nanoparticles in the literature [30,31]. It was important to determine the effects of using low and high concentrations of SPIONs relative to the baseline toxicity level reported in the literature. 3D bone constructs were designed in Autodesk Fusion 360 CAD software (Autodesk, Inc. Mill Valley, CA, USA) and converted to the standard STL file format for printing. 

Two different geometries were used for the experiments: a disc and a taller cylinder construct. The disc had a diameter of 3.5 mm and a height of 0.9 mm. The taller cylindrical constructs also had a diameter of 3.5 mm but was extended to have a height of 5 mm. The constructs also had an average pore size ranging from 0.6 mm to 0.75 mm and featured a wall thickness of 0.25 mm between each pore (according to the CAD design). The disc constructs were used for in vitro experiments, including cell culture, µCT, and antibacterial activity analyses. The cylindrical constructs were used for in vivo experiments in the rat model. Both construct designs were used for mechanical testing and material characterization studies. All constructs were printed at room temperature utilizing a six-axis robotic arm 3D bioprinter (BioAssemblyBot, Advanced Solutions Life Sciences, Louisville, KY, USA). Layer thickness was designed to be 0.2 mm to ensure the fusion of each layer. Prior to cell seeding, samples were processed post-print to rinse constructs of solvent materials and to sterilize.

### 2.2. Microscopy, Spectroscopy, and Thermogravimetric Analyses of the 3D Bone Constructs

A variety of nanoscale characterization studies were conducted on scaffolds to assess incorporation of SPIONs in HB structure. Field-emission scanning electron microscopy (FESEM) images were obtained using MERLIN Compact and SUPRA 55VP Zeiss instruments equipped with an energy-dispersive X-ray spectroscopy (EDX) detector. FESEM studies on scaffolds with and without SPIONs were used to verify the presence of nanoparticles in the SPION-loaded HB scaffolds. Both FESEM and EDX elemental mapping were used to examine the effect of SPION incorporation on the structure of HB constructs. X-ray photoelectron spectroscopy (XPS) was acquired using an Al Kα source (Sigma Probe, Thermo VG Scientific). XPS analysis was used to precisely examine the presence of the SPIONs and all the other constructive compounds (P 2p, C 1s, Ca 2p, and Fe 2p) in the printed scaffolds. Raman was performed on a LabRAM HR Evolution, HORIBA instrument. To further study the role of SPIONs on the structure of HB constructs, we conducted TGA studies. The thermogravimetric analysis (TGA) results were obtained using a TGA Q5000 IR machine (TA Instruments, New Castle, DE, USA).

### 2.3. Mechanical Testing

The AR-G2 Magnetic Bearing Rheometer (TA Instruments, New Castle, DE, USA) testing system equipped with a 50 N load cell was utilized to conduct compression testing of the HB constructs. Cylindrical scaffolds were tested in both axial and radial directions, whereas the disc scaffolds were tested only in the axial direction. Loading rate was set at 0.01 mm/s. Moduli and stiffness values were calculated from the initial 15% linear region of stress–strain curves. Nanoindentation tests were carried out using a NanoTest instrument (Micro Materials Ltd. Wrexham).

### 2.4. Computed Tomography (CT) Analysis

Microcomputed tomography (µCT) qualitative assays were performed in the in vitro experiments to determine the effect of SPION incorporation on mineralization and bone formation at a macroscopic level. Traditional mineralization tests such as Alazarin Red was not applicable for these 3D printed constructs due to their inherent high calcium phosphate content (~90 wt %). µCT analyses were performed according to the current guidelines for the assessment of microstructure of the 3D constructs. For the in vitro imaging, bioprinted 3D constructs were scanned using a µCT 40 (Scanco Medical AG, Bassersdorf, Switzerland) at 55 kVp tube voltage, 250 ms integration time, and 20 μm isotropic voxel size. The region of interest (ROI) selected for analysis consisted of 70 transverse CT slices representing the entire volume of the construct. 3D reconstructions were created by stacking the ROIs from each 2D slice and then applying a gray-scale threshold and Gaussian noise filter. The µCT slices (70) were subjected to a 3D Gaussian low-pass filter (σ  =  0.8, support = 1.0) to remove noise, and a fixed threshold of 150. Bone volume (BV) and fractional bone volume (bone volume/tissue volume; BV/TV) were calculated. For the animal study, sequential transaxial images for the middle third of the femur were obtained at 55 kVp tube voltage, 250 ms integration time, and 30μm isotopic voxel size. 

### 2.5. Cell Culture—Cell Seeding of Printed Constructs

In vitro cell culture studies were performed to assess cell viability and mineralization potential of embryonic murine C3H10T12 cells and human-patient-derived osteoblast-like HBO cells. A mouse embryonic fibroblast cell line, C3H10T12 cells, Clone 8 (ATCC: CCL-226) were seeded in T75 flasks (Sigma, Z707546). The cells were maintained and passaged, according to manufacturer’s instructions, using alpha-MEM (Gibco, 12571071) + 10% FBS (Atlanta Biologics, S11150) + 1% antibiotics (penicillin/streptomycin) (Gibco, 15240-062). A human bone osteoblast-like (HBO) cell line was isolated from fibular donor bones obtained under an IRB-approved protocol. The bone was broken into pieces and first subjected to digestion, twice, using single-digest media (serum-free Dulbecco’s modified Eagle’s medium (DMEM) + antibiotics (100 µg/mL Primocin) + 0.04% trypsin + 0.1 mg/mL collagenase A) while shaking for 20 s every 10 s for 1 h. DMEM (Corning, 10-013-CV) + 10% FBS + Primocin (Invivogen, ANTPM1) was added to the digested bone, which was then left undisturbed until cells were observed to dissociate from the bone matrix and attach to the plate. HBO cells were expanded and maintained in DMEM + 10% FBS + Primocin until use.

#### 2.5.1. Osteogenic Cultures

Upon reaching 70% confluency in the T75 flasks, C3H10T12 or HBO cells were trypsinized and reseeded into 3D printed constructs in 48-well tissue culture plates (Sigma, St Louis, MO, USA) at a density of 100,000 cells/construct. For this purpose, 3D constructs, incorporating 0 (control), 60, and 200 µg/mL of SPIONs, were inserted into individual wells at a sample size of 6 for each condition. The capacity of the cells to mineralize the surrounding matrix was tested by providing osteogenic media (OM): α-MEM (for C3H10T12) or DMEM (for HBO) containing 10% FBS, 1% penicillin/streptomycin, 100 μg/mL ascorbic acid (Fisher, A62-500), and 5 mM β-glycerophosphate (G9422-50G, Sigma, St Louis, MO, USA). Half-feeds were given every 2 days. Within this experiment, the cellular activity of C3H10T12 and HBO cells was measured utilizing an alamarBlue assay (BioRad, Richmond, CA, USA) at days 3, 7, 10, 14, and 17. On day 21 of culture, cellular constructs were either fixed using 50% ethanol for 15 min at 4 °C and the 3D constructs were then subjected to µCT analysis or constructs were placed in cell lysis buffer provided in the Qiagen RNeasy kit (74104, Qiagen, Hilden, Germany) and RNA was isolated from cells embedded in the construct according to manufacturer’s protocol provided in the kit.

#### 2.5.2. Macrophage Cultures

Bone-marrow-derived macrophages (BMDMs) were isolated and prepared from C57/BL6 mice femur and tibias. Red blood cells were lysed 30 s using ACK lysing buffer, and then the remaining cells were cultured in complete RPMI media supplemented with 50 ng/mL of recombinant mouse M-CSF for 7 days at 5 million cells in a BD Falcon tissue culture dish. On day 3, nonadherent cells and 75% of culture media were exchanged with fresh media. On day 7, all cells were collected and plated in full media, serum-free media, 10% FBS serum-containing media, 10% HS serum-containing media, 50% FBS serum-containing media, and 50% HS serum-containing media followed up with the addition of SPION at a concentration of 2.73 mg Fe ml^−1^.

### 2.6. Bacteria and Macrophage Culture—Antibacterial Activity Assays

Bacteria and macrophage culture were performed to simulate a bacterial infection in vitro. Constructs (*n* = 6) including the 0 (HB control), 60, and 200 µg/mL of SPIONs conditions were cultured with or without human THP-1 macrophages (ATCC TIB-202) in DMEM (Gibco 11885092) supplement with 10% FBS (Corning 35-010-CV) as a media (no antibiotics) up to 3 days prior to bacteria seeding. Green-fluorescent-protein (GFP)-labeled *Staphylococcus aureus* (*S. aureus*) (ATCC 43300 transformed with pTRKH3-ermGFP (Addgene plasmid #27169)) were cultured onto 3DP bone constructs with and without SPIONs up to 24 h to determine remaining bacteria levels. The Bio-Rad ChemiDoc^™^ MP Imaging System (BioRad, Richmond, CA, USA) was used to retrieve and map the remaining fluorescence of GFP-labeled *S. aureus* on 2D controls and 3DP constructs. Images were processed in ImageJ to quantify levels of GFP intensity of each sample concentration. 

### 2.7. Animals

The experiments were approved by the Institutional Animal Care and Use Committee (IACUC) at Beth Israel Deaconess Medical Center (BIDMC). Sixteen adult male and female Sprague Dawley rats (aged 13 weeks) were used for this study. Rats were subjected to surgery to create a 5 mm midshaft femoral bone defect, divided into two treatment (*n* = 12) and control (untreated) groups (*n* = 4). In the treatment group, 3D bioprinted cylindrical HB scaffolds (5 mm height × 3.5 mm diameter) were inserted into the defect site. In the control group, the defect site was left empty. Unrestricted ambulation was allowed after surgery, and pain was controlled using sustained-release buprenorphine, injected subcutaneously at a dose of 1.2 mg/kg. At 2, 8, and 12 weeks post-surgery, animals were euthanized. The operated site was visually inspected for scaffold stability. Specimens were then examined qualitatively for new bone formation using μCT imaging and histological studies (*n* = 4 per group). All experiments were performed in accordance with relevant guidelines and regulations. A total of 16 animals were used in this study. Due to the gender effect on femoral defect healing in rats, both male and female rats were used in this pilot study. Male and female genders were distributed equally in both groups at all time points. Four animals were used to test the fracture healing without scaffold at 12 weeks postoperative, and 12 were used to study the effect of the scaffold on the bone defect healing at 2, 8, and 12 weeks (4 animals per each time point).

### 2.8. In Vivo Application of Bioprinted HB Scaffolds

Anesthesia was induced using isoflurane at 5% in an induction chamber and then maintained at 2.5% via nose cone. Following anesthesia, animals were weighed for baseline weight and then placed on a heating pad for further preparation. The skin of the right hind limb was shaved and disinfected. Sterile surgical drapes were used to cover the animal, leaving the surgical site exposed. A skin incision between the greater trochanter and the knee joint followed by incision of the superficial fascia was used to achieve a lateral approach to the thigh. The intermuscular plane between the vastus lateralis and the biceps femoris muscles was separated to expose the femoral shaft. A radiolucent, weight-bearing polyether ether ketone (PEEK) plate (Special Designs, Suffern, NY, USA) was secured to the exposed femur with four bicortical 0.9 mm threaded k-wires (MicroAire, Charlottetown, VA, USA) into predrilled holes. Using a 0.22 mm Gigli wire saw (RISystem, Davos Platz, Switzerland), a bone defect was then achieved via two cuts at the midshaft of the femur spaced at 5 mm distance, guided by premade grooves at the bone facing surface of the plate. After removal of the 5 mm bone segment, the defect site was thoroughly irrigated with sterile normal saline solution to remove bone debris; then, the bony defect was either packed with a scaffold (treatment group) or left empty (control group). The muscular layer was then approximated with absorbable sutures and the skin was closed using skin clips. 

This pilot study investigated the effect of packing a critical size femur defect in a rat model using a bioprinted SPION-laden scaffold. Due to the difficulty of estimating the effect size, the traditional sample size calculation was impossible. Instead, we used the resource equation method, which is commonly used in exploratory animal studies [32].

### 2.9. Histological Analysis

Harvested bone specimens were cleaned of the attached soft tissue and fixed immediately in 10% neutral buffered formalin at room temperature for 48 h. After dissection, the fixed specimens were dehydrated in graded ethanol for 1 week. The nondecalcified samples were infiltrated and embedded in methyl methacrylate (85%) and dibutyl phthalate (15%) (Sigma-Aldrich, St. Louis, MI, USA). Nonconsecutive longitudinal sections (5 µM) were obtained at the center of implanted tissue with a high impact microtome (RM2255, Leica) (*n* = 3 per sample, per group). Identification of cellular and mineralized components of bone was performed on Goldner’s trichrome and von Kossa stained sections.

### 2.10. Statistical Approaches

Statistical analysis was performed using JMP (JMP Statistical Discovery from SAS, Cary, NC, USA) software. Significant differences were determined with one-way ANOVA or two-way ANOVA if applicable. A post hoc Tukey–Kramer test was performed for multiple comparisons and a *p*-value of < 0.05 was considered statistically significant (* *p*-value < 0.05, ** *p*-value < 0.01, **** *p*-value < 0.0001). Least square means connecting letters reports were also used to show significant differences between multiple comparisons. Levels not connected by the same letter are significantly different. Levels connected by the same letter are not significantly different.

## 3. Results and Discussion

### 3.1. 3D Bioprinting of Damage-Specific SPION-Loaded Bone Constructs—Material Characterization of Bioprinted Scaffolds

**Figure 1:**
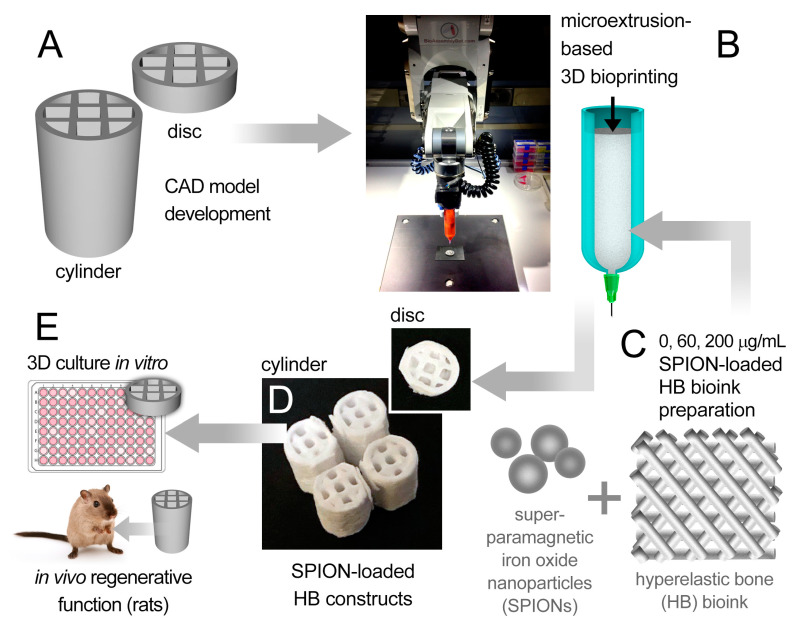
**Schematic summary of the experimental method used in this study**. (**A**) Two computer-aided design (CAD) models (disc and cylinder) were designed and bioprinted using a six-axis robotic arm bioprinter (**B**). A hyperelastic bone (HB)-based bioink (**C**), loaded with varying doses of superparamagnetic iron oxide nanoparticles (SPIONs) (0, 60, and 200 µg/mL), was used to print the disc and cylindrical bone-like scaffolds (**D**). (**E**) Disc HB constructs were assessed in vitro and cylindrical HB constructs were tested in vivo for their potential to generate bone tissue.

**Figure 2:**
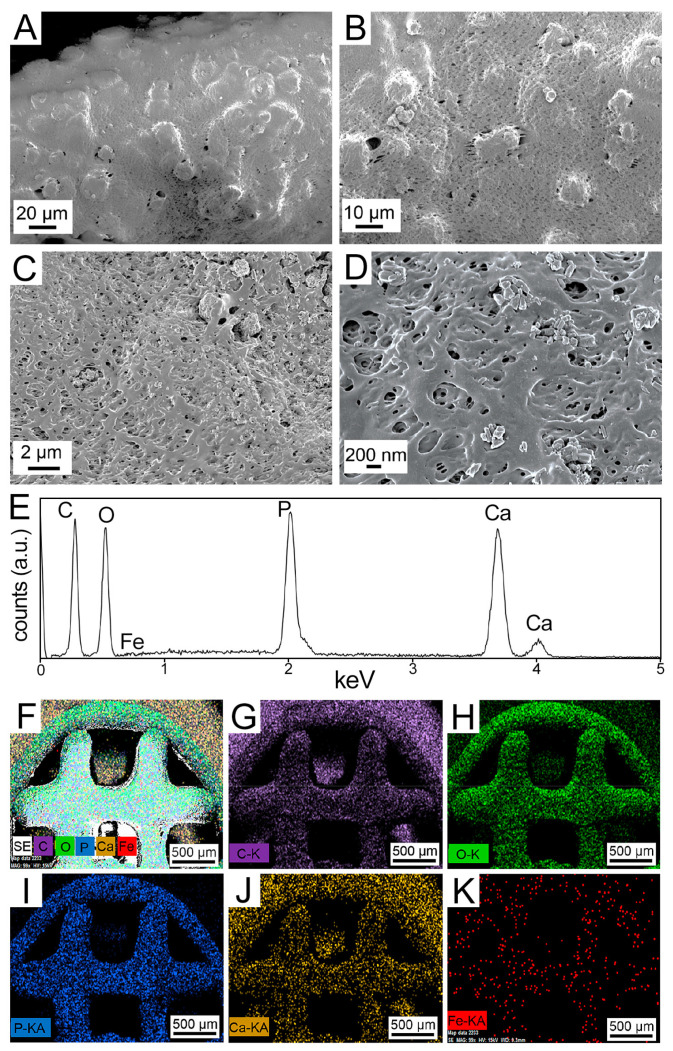
**Material characterization analyses conducted on bioprinted 60 µg/mL SPION-loaded HB scaffolds**. (**A–D**) Field-emission scanning electron microscopy (FESEM) images of the SPION-loaded 3D bioprinted HB scaffold at varying magnifications. (**E–K**) Energy-dispersive X-ray spectroscopy (EDX) spectrum of the HB-SPION scaffold (**E**), along with the EDX maps of (**F**) secondary electrons, (**G**) carbon (C), (**H**) oxygen (O), (**I**) phosphorous (P), (**J**) calcium (Ca), and (**K**) iron (Fe) in the printed constructs.

Two designs of 3D porous bone structures were bioprinted and tested in the in vitro (HB discs) and in vivo (HB cylinders) studies (Figure 1). The damage-specific HB cylinders were bioprinted for the rat studies, based on the precise geometry (CAD model) that was used by the surgery team to generate the femoral bone defects. The HB discs were used for all in vitro analyses. Field-emission scanning electron microscopy (FESEM) images of printed discs (Figure 2A–D) revealed that the SPION-loaded scaffolds had a smooth hierarchical assembly structure, whereas the SPION-free constructs appeared to have a rough and defaced surface (Figure S1A–C). The energy-dispersive X-ray spectroscopy (EDX) spectra ascertained the presence of carbon (C), oxygen (O), phosphorous (P), and calcium (Ca) in the SPION-loaded (Figure 2E) and SPION-free (Figure S1D) scaffolds. The EDX elemental mapping (Figure 2F–K) further demonstrated a discrete distribution of SPIONs (i.e., Fe element), and other selected elements, uniformly dispersed throughout the scaffold. This was further confirmed with the complementary pertinent high-resolution FESEM images and EDX map (Figure S2). 

**Figure 3:**
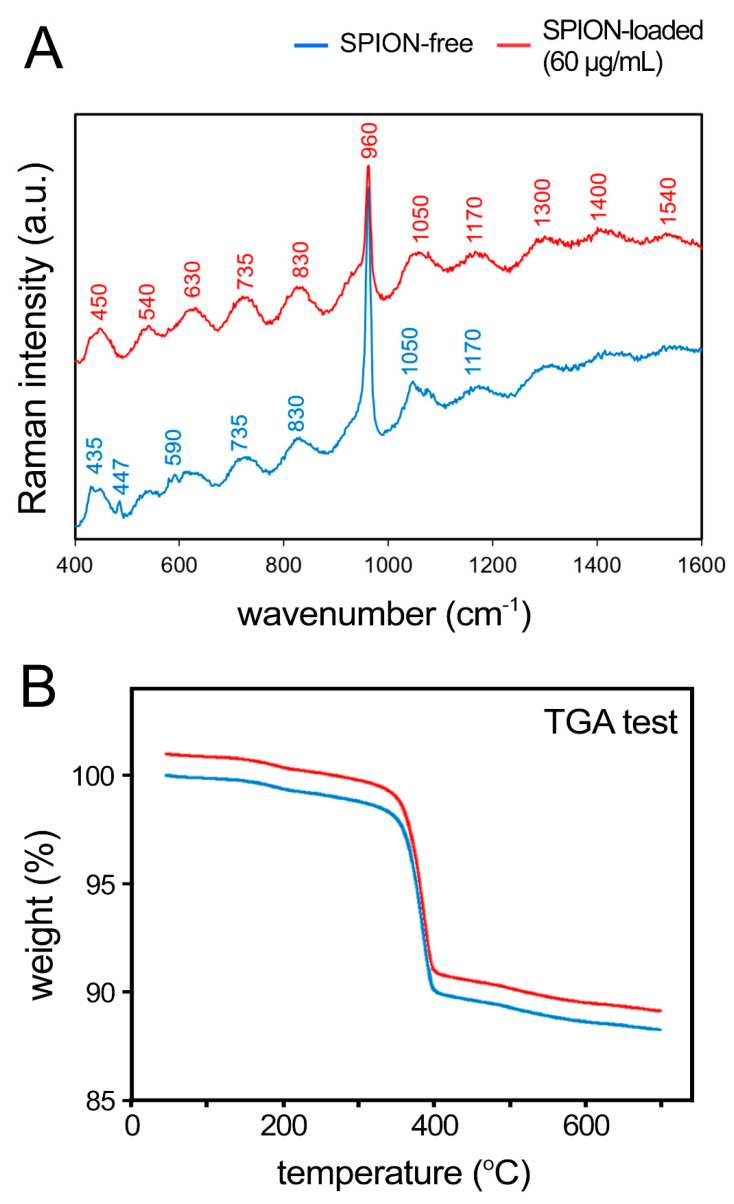
**Raman analysis and thermogravimetric analysis (TGA) of 3D bioprinted HB scaffolds.** (**A**) Raman spectra of the SPION-free (blue) and SPION-loaded (red, 60 µg/mL) scaffolds. (**B**) TGA of both empty and SPION-loaded constructs.

X-ray photoelectron spectroscopy (XPS) analysis also confirmed the existence of the all expected components in both SPION-loaded (Figure S3) and SPION-free (Figure S4) scaffolds. As expected, however, only spectra of the SPION-loaded scaffolds demonstrated the existence of elemental Fe (Figure S3). In addition, Raman spectroscopy was used for the detection of the SPIONs within the scaffolds. The specific wavenumbers of 1300, 1400, and 1540 cm^−1^ for SPIONs were presented (Figure 3A). A similar stepwise decomposition of both SPION-loaded and SPION-free scaffolds was identified using thermogravimetric analysis (TGA) (Figure 3B). 

### 3.2. Mechanical Characterization of Bioprinted Scaffolds

Axial loading of the printed disc HB scaffolds demonstrated that both SPION-free and SPION-loaded constructs retain a high degree of elasticity in both cylindrical (Figure S5A) and disc (Figure S5B) scaffolds. While SPION-free constructs in cylindrical geometry showed a significantly greater modulus than that in the disc scaffolds, incorporation of SPIONs resulted in a significant decrease in the modulus of both groups (Figure S5C). By addition of nanoparticles, the compressive modulus of tall cylinders significantly decreased from 10.21 MPa in SPION-free scaffold to 7.22 and 7.70 MPa in scaffolds containing 60 and 200 µg/mL SPIONs, respectively. Modulus decreased more notably in the disc scaffolds from 3.70 MPa in SPION-free samples to 0.59 and 0.50 MPa in scaffolds containing 60 and 200 µg/mL SPIONs, respectively. The range of elastic modulus obtained in the cylindrical scaffolds (7–10 MPa) was within the optimal range of modulus reported by other studies to promote bone repair [33,34]. Reduced mechanical properties in SPION-laden constructs can be attributed to the compositional nature of the bioink material. Since the used bioink is a composite material containing 90% hydroxyapatite with the remaining mostly composed of PLGA, the SPIONs may allow for the formation of microcracks, diminished fusion of printed layers, or bulk material instability. This is mainly due to the fact that the SPION-loaded bioink solution is a colloidal matrix and no chemical bonding occurs when preparing the ink [35,36]. It has been also previously reported that the geometry and porosity of 3D printed HB constructs can significantly affect the ultimate mechanical behavior [28].

We compared axial versus radial loading in the cylindrical SPION-free HB constructs (Figure S6). Results showed anisotropic mechanical properties in the printed scaffolds, with significantly greater modulus and stiffness values in the axial versus radial orientation (Figure S6C,D). The hardness and elastic modulus of the printed scaffolds were also assessed using the nanoindentation method, demonstrating mechanical properties improvement in the SPION-loaded scaffolds in comparison to the SPION-free group (Figure S6D). In contrast to the macroscale compressive testing of cylindrical HB constructs, nanoscale indentation of HB constructs showed an increase in elastic modulus of scaffolds. This could be attributed to the differences often observed in nanoscale, local deformation versus bulk, global deformation in nanostructured materials, which results in greater mechanical enhancement in smaller scales [37]. 

### 3.3. In Vitro Cell Viability and Growth in Bioprinted HB Scaffolds

**Figure 4:**
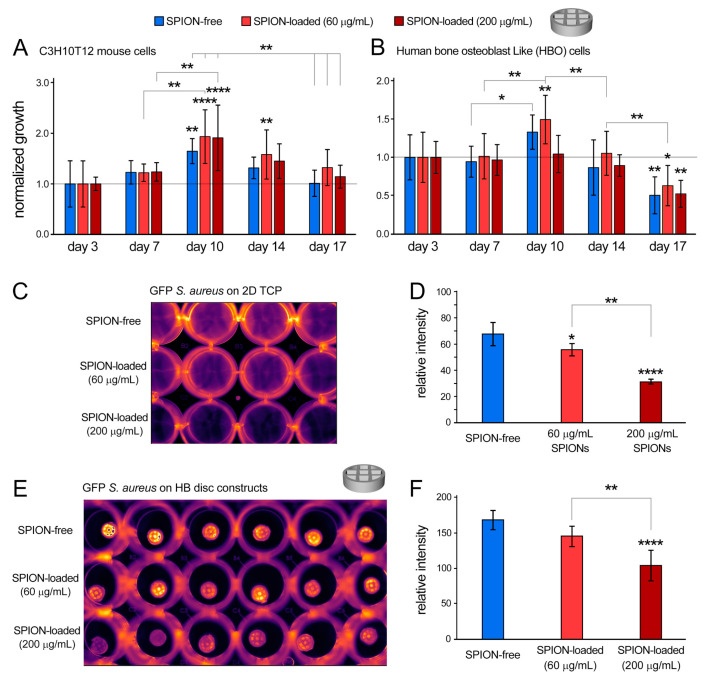
**Characterization of cellular and bacterial response to bioprinted HB constructs in vitro.** (**A**,**B**) Cellular growth (normalized to day 3) for C3H10T12 mouse cells (**A**) and human bone osteoblast (HBO) cells (**B**), measured by the noninvasive AlamarBlue assay for 17 days of in vitro culture. (**C–F**) Bacteriostatic effects of SPION in 2D culture (**C**,**D**) and in SPION-loaded HB constructs (**E**,**F**) were evaluated by culturing GFP+ *S. aureus* onto scaffolds for 24 h (**C**,**E**) and measuring fluorescence signals (**D**,**F**). * *p* < 0.05, ** *p* < 0.01 and **** *p* < 0.0001.

Along with providing mechanical integrity, HB materials have been also shown to support the culture of human mesenchymal stem cells, while expressing pro-osteogenic factors [28]. To further assess biocompatibility of the developed bioink and examine potential cytotoxicity of incorporated nanoparticles, C3H10T12 and human bone osteoblast-like (HBO) cells were cultured onto the printed HB discs for up to 17 days. The C3H10T12 cells are a fibroblast cell line that can take on osteoblast phenotype in bone-inductive environments [38]. Experimental groups included the control SPION-free group and SPION-loaded (60 and 200 µg/mL) scaffolds (Figure 4 and Figure S7). All conditions were normalized to their respective day 3 values. AlamarBlue results demonstrated that both cell types remained viable and proliferated during the 17-day 3D culture period (Figure 4A,B). The CH3H10T12 cell line showed significant increases in growth from day 3 to days 10 and 14 (*p*-value < 0.0001). A significant drop in growth was observed from day 10 to days 14 and 17 (*p*-value < 0.0001). Notably, the 60 µg/mL SPION-loaded group maintained statistically higher viability and growth than that of the SPION-free control group at most time points (*p*-value = 0.0282). In addition, the C3H10T12 line maintained an average cellular growth above the day 3 baseline throughout the experiment, in contrast to the HBO line (Figure 4A,B). 

The HBO cell line showed a growth trend consistent with that observed for the C3H10T12 line. There was a significant increase in cell growth from day 3 to day 10 (*p*-value < 0.0001). Significant decreases in cellular growth were obtained from day 3 to 17 and from day 10 to days 14 and 17 (*p*-value < 0.0001). Consistent with the mouse cell line, the 60 µg/mL SPION-loaded group appeared to keep statistically higher viability of HBO cells than the 200 µg/mL SPION-loaded group (*p*-value = 0.0042) (Figure 4B). These results suggest that the 60 µg/mL SPION-loaded constructs would be an optimal group to support cell viability and growth to be used for the in vivo study.

The decline at later time points in 3D culture (day 17) may be attributed to limited oxygen and nutrient diffusion with the relatively thick 3D constructs that, at prolonged culture, could affect cell viability and growth [39,40]. Furthermore, a degree of variation in cellular metabolic activity is frequently observed in these noninvasive (AlamarBlue) assays, which may not directly reflect on lower cell number, but indicates slower cellular metabolism [41].

We next performed qPCR analysis of RNA collected from SPION-free (control) and SPION-loaded HB constructs and analyzed multiple markers of mineralization and osteogenesis in both mouse and human cells (Figure S8). The results demonstrated significant increases in the expression of mouse osteopontin (mOPN), human osteocalcin (hOCN), and human alkaline phosphatase (hALP) by addition of the nanoparticles to the HB constructs (Figure S8A,D,E). This is while expression of mouse osteocalcin (mOCN) and alkaline phosphatase (mALP) were significantly reduced in SPION-loaded constructs (Figure S8B,C). These results suggest a cell-type-specific response to bioprinted HB constructs that are laden with the SPIONs. The 60 µg/mL SPION-loaded group exhibited greater potential for osteogenesis and mineralization, in comparison to the 200 µg/mL group, and, therefore, was selected for the in vivo assays. 

### 3.4. In Vitro Computed Tomography (CT) Imaging of Bioprinted SPION-Loaded Constructs

Microcomputed tomography (µCT) analysis was performed to visualize the 3D architecture of bioprinted implants and to assess the bone volume of HB constructs following the in vitro cell culture (21 days) (Figure S9). Acquired CT images clearly revealed the internal porous structure of the printed constructs, which closely corresponded to the CAD designs (Figure 1A) and were stable following 21 days of in vitro culture (Figure S9A,B). The control SPION-free and the 60 µg/mL SPION-loaded groups showed no significant difference in the bone volume after the 3-week culture (Figure S9C), indicating that incorporating the nanoparticles did not elicit a major effect on the osteogenic capacity of bioprinted HB constructs. 

### 3.5. Bacteriostatic Activity of Bioprinted HB Scaffolds

Implant-associated infection is still considered to be one of the most serious and common complications in implant surgeries [27]. Therefore, antibacterial characteristics of biomaterials would be an important factor in their clinical applications. Among different types of nanoparticles, SPIONs have attracted a great deal of attention for bone regeneration due to their distinctive antibacterial [42,43] and biocompatibility [43] properties. However, improving antibacterial properties of bioinks and printed implants through incorporation of nanoparticulate materials have not been reported. We have previously demonstrated the critical intrinsic properties of SPIONs and their unique capacity in removing bacterial biofilms in the presence of macrophages [42,43]. In agreement with these findings, here we found that addition of 60 and 200 µg/mL of SPIONs to the two-dimensional (2D) culture significantly reduced the activity of *Staphylococcus (S.) aureus*, the most common bacteria associated with implant infection/rejection [44], as measured by the GFP signal after 17 h of culture (*p* < 0.05 and < 0.0001 for 60 and 200 µg/mL, respectively) (Figure 4C,D). Increasing the SPION concentration from 60 to 200 µg/mL increased the bacteriostatic activity (*p* = 0.0008). To further assess the bacteriostatic effect of SPIONs, they were incorporated into the HB bioink at 60 and 200 µg/mL concentrations. Analogous to the 2D control study, introducing 200 µg/mL of SPIONs to the bioprinted HB constructs resulted in a significant (~38%) decrease in GFP-labeled *S. aureus*, in comparison to the constructs with no SPIONs (*p* < 0.0001) (Figure 4E,F). Furthermore, consistent with the 2D controls, incorporating 200 µg/mL of SPIONs increased antibacterial activity in comparison to the 60 µg/mL SPION-loaded group (*p* = 0.0021). The 60 µg/mL group, however, exhibited the most optimal in vitro cell response and, therefore, was selected to continue this study in vivo. 

### 3.6. In Vivo Regenerative Capacity of Bioprinted Damage-Specific HB Constructs

Alarming signals of suboptimal clinical success for the current scaffold systems in healing large bone fractures raises several questions with regards to the causes of failure and highlights the need to develop alternative technologies. Here, we used a multifunctional and highly tunable yet straightforward system that can pave the way for successful clinical translation of personalized regenerative approaches in healing large fractures. 

**Figure 5:**
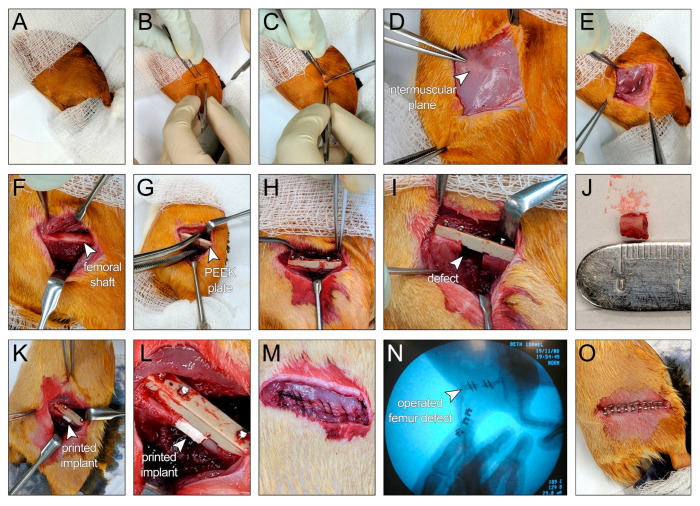
**Surgical procedure to generate 5 mm midshaft femoral bone defects in Sprague Dawley rats.** (**A**) The skin of the right hind limb was prepared, and surgical drapes were placed to cover the animal, leaving the surgical site exposed. (**B**) A line between the greater trochanter and the knee joint was identified before incision. (**C**) Skin incision was made between the greater trochanter and the knee joint. (**D**) The white line representing the intermuscular plane between the vastus lateralis and the biceps femoris muscles was identified before fascial incision. (**E**) The intermuscular plane between the vastus lateralis and the biceps femoris muscles was separated. (**F**) Femoral shaft was exposed. (**G**) The polyether ether ketone (PEEK) plate was temporarily secured to the exposed femur using surgical forceps. (**H**) The PEEK plate was permanently secured to the femur with four bicortical 0.9 mm threaded k-wires into predrilled holes. (**I**) A 5 mm bone defect was achieved via two cuts at the midshaft of the femur, spaced at a 5 mm distance, using a Gigli wire saw and guided by premade grooves at the bone facing surface of the plate. (**J**) A 5 mm bone segment after removal from the defect site. (**K**,**L**) The defect site was filled with the bioprinted HB scaffold. (**M**) The muscular layer was approximated with absorbable sutures (Vicryl 5-0). (**N**) X-ray image for the operated femur. (**O**) The skin was closed using skin clips.

**Figure 6:**
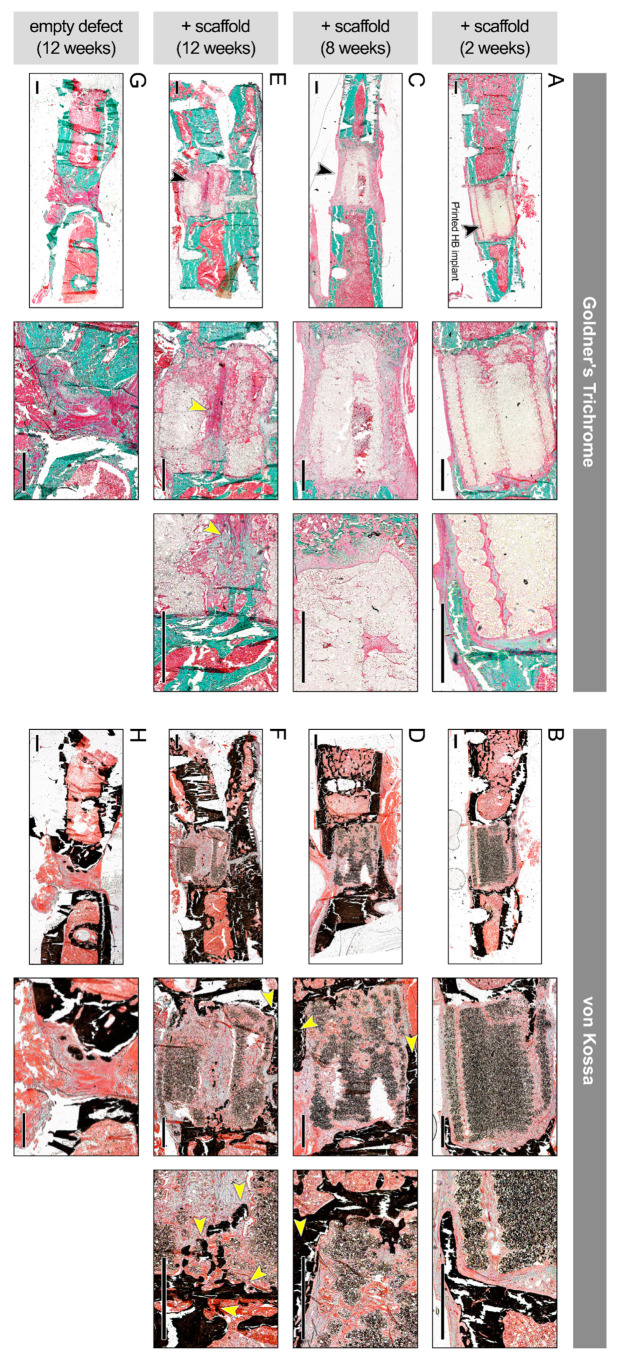
**In vivo regenerative effect of bioprinted HB scaffolds in a rat femoral fracture model.** Histological images stained with Goldner’s trichrome stain (left), (**A**,**C**,**E**,**G**) and von Kossa stain (right), (**B**,**D**,**F**,**H**), obtained from weeks 2, 8, and 12 posttreatment, at two or three different magnifications (left to right). (**A**,**B**) Defects treated with 60 µg/mL SPION-loaded HB constructs showed early mineralization at 2 weeks. Arrow points to the HB implant inserted within the femoral shaft. (**C**,**D**) At 8 weeks, there was cellular infiltration and nonmineralized soft tissue in the middle region, as well as mineralization at periphery (yellow arrows). Black arrow points to the grafted HB scaffold. (**E**,**F**) Significant soft tissue in the middle region (yellow arrows) and peripheral bridging were present at 12 weeks. (**G**,**H**) Empty defect without scaffolds, used as control group, failed to bridge at 12 weeks and showed significant fibrosis. Scale bars denote 1 mm.

The effect of bacteriostatic, SPION-loaded HB scaffolds on bone regeneration was tested in vivo by generating a midshaft femoral bone defect in Sprague Dawley rats (Figure 5). The bone defects were created precisely based on the geometrical (CAD) design that was used to print the HB cylindrical constructs; thus, the HB implants closely fitted within the femoral defect, with no gap between the implant and host tissue (Figure 5K–L, Figure S10). Particularly, 12 weeks post implantation, harvested tissues showed adequate engraftment and integration of HB scaffolds into the host femur tissue, with no evidence of infection (Figure S10B). Histological analysis on bone defects treated with 60 µg/mL SPION-loaded HB constructs demonstrated early mineralization along the periphery of the scaffolds within 2 weeks (Figure 6A,B). A small amount of cellular infiltration into the scaffolds was observed at this time point. Mineralization along the periphery of the scaffold was enhanced at the 8-week time point (Figure 6C,D). By 8 weeks, cells were able to infiltrate into the scaffold, particularly in the middle region, where apparent nonmineralized soft tissue was observed. Defects were bridged along the periphery of the scaffold at the 12-week time point, suggesting an osteoconductive effect of the scaffold (Figure 6E,F). Qualitatively, more cellular infiltration was observed at this time point, with greater amounts of soft tissue and some early signs of mineralization within the scaffold. Histological analysis also demonstrated the layered porous structure of HB scaffolds, which gradually fused together to form a more contiguous, integrated tissue from weeks 2 to 12. Untreated defects failed to bridge and showed significant fibrosis within the defect at 12 weeks, as examined via Goldner’s trichrome and von Kossa staining (Figure 6G,H). 

**Figure 7:**
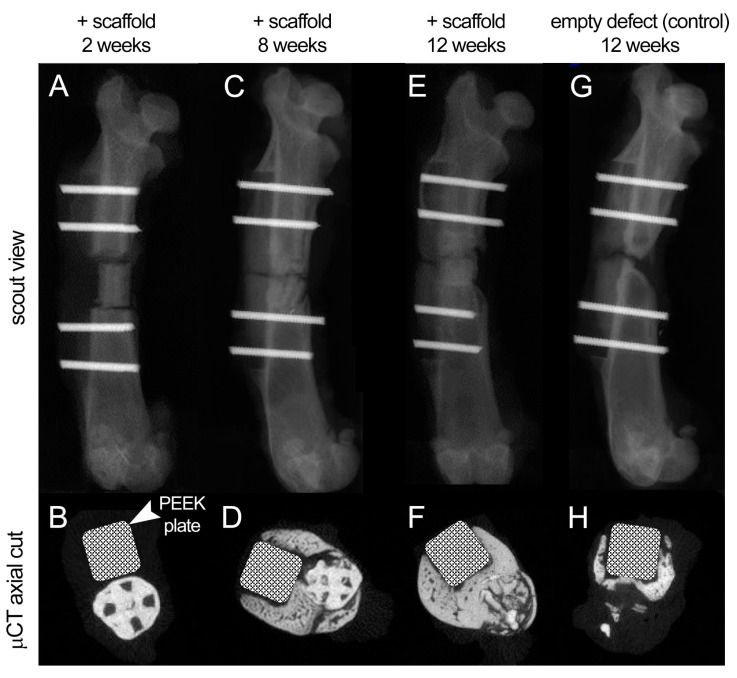
**Scout views and µCT axial cuts of the rat femur at 2, 8, and 12 weeks of follow-up.** (**A**,**B**) At 2 weeks after surgery, the scaffold was well aligned within the defect site with minimal new bone formation at the bone ends. The structure of scaffold was well maintained, and no signs of mineralized callus was evident. (**C**,**D**) At 8 weeks, there was more evident bone formation around and within the scaffold with disruption of the scaffold wall at the plate side. (**E**,**F**) At 12 weeks, complete bone bridging was achieved and fading of the scaffold structure, which was replaced by mineralized bone. (**G**,**H**) The control group, with no scaffold, failed to achieve bone bridging at 12 weeks after surgery with scarce bone formation at the plate side.

These finding were further validated by the qualitative μCT analysis of treated animals (Figure 7, Figures S11 and S12). The cylindrical scaffolds were well retained and aligned within the defect sites, with minimal gaps, confirming the adequate fitting of damage-specific implants. There was minimal bone formation at the two bone ends at 2 weeks after surgery and no signs of mineralized callus. (Figure 7A,B and Figure S11A–D). At 8 weeks, there was evident new bone formation around and within the scaffold, along the whole length of the defect, with signs of scaffold wall disruption more noticeably at the plate side (Figure 7C,D and Figure S11E–H). At 2–8 weeks, bone formation was more evident in male rats (Figure S11E–H). At 12 weeks, the scaffold-treated group achieved complete bone bridging, more evidently in male rats, as well as evident fading (degradation and integration with host tissue) of the scaffold structure which was being replaced by mineralized bone (Figure 7D,E, Figure S12A–D), while the control group failed to do with scarce bone formation at the defect site (Figure 7F,G, Figure S12E–H). 

## 4. Conclusions

We developed a novel 3D bioprinted nanoparticle-functionalized bone scaffold system with enhanced bacteriostatic properties and a complex, porous, and customized structure. This platform can be used as an in vitro model for 3D culture and analysis or for tissue regenerative therapies of nonhealing large bone fractures as patient and damage-specific implants with significantly diminished risk of infection or rejection. Such functionalized bioprinted implants could be customized for a broad variety of hard and soft tissue regeneration. The extent and diversity of the in vitro characterization and in vivo analyses conducted in this study establish a unique and effective precision medicine approach, demonstrating how nanoscience and biomaterials science, bioengineering and biofabrication, and surgical and clinical procedures could synergize to establish new regenerative therapies. Our study did not investigate concentrations of SPIONs in the bioprinted constructs that fall between or outside of the two experimental groups (60 and 200 µg/mL). Investigating the effect of varied particle concentration within the bioprinted constructs could be of great significance in determining an optimal concentration to elicit a maximal regenerative function while avoiding major cytotoxic effects. Furthermore, coupling the conducted in vivo assays with magnetic resonance imaging and more in-depth analysis of infection and/or inflammation processes could be a direction for the future works.

## Supplementary Materials

The following are available online at https://www.mdpi.com/article/10.3390/polym13071099/s1, Figure S1: Material characterization analyses conducted on bioprinted SPION-free (empty) HB scaffolds. A-C: FESEM images of the SPION-free bioprinted HB scaffold at varying magnifications. D-H: EDX spectrum of the HB scaffold (D), along with E: the EDX maps of 1: carbon (C), 2: oxygen (O), 3: phosphorous (P), and 4: calcium (Ca) in the SPION-free printed constructs. Figure S2: A and B: Complementary low and high magnification FESEM images of the 60 µg/mL SPION-loaded HB constructs, together with C: the EDX maps of 1: carbon (C), 2: oxygen (O), 3: phosphorous (P), 4: calcium (Ca), and 5: iron (Fe) in the SPION-loaded scaffolds. Figure S3: XPS analysis of bioprinted 60 µg/mL SPION-loaded HB constructs. A: survey scan, B: P 2p, C: C 1s, D: Ca 2p, and E: Fe 2p of the printed nanoparticle-loaded HB scaffold. Figure S4: XPS analysis of bioprinted SPION-free HB constructs. A: survey scan, B: P 2p, C: C 1s, D: Ca 2p, and E: O 1s of the printed HB scaffold. Figure S5: Mechanical characterization of bioprinted HB constructs. A-B: Compressive mechanical testing of the SPION-free (blue) and SPION-loaded (bright and dark red at 60 and 200 µg/mL, respectively) scaffolds in cylindrical (A) and disc (B) shape. C: Compressive modulus HB constructs measured at 15% strain. * *p* < 0.05, ** *p* < 0.01, *** *p* < 0.001 and **** *p* < 0.0001. Figure S6: Additional mechanical characterization of bioprinted HB constructs. Stress-Strain (A) and Force-displacement (B) curves of cylindrical HB constructs, obtained from compressive mechanical testing in axial versus radial directions demonstrated distinct behavior. C-D: No significant differences were observed in modulus (C) and stiffness (D) values of cylindrical constructs in axial versus radial directions. E: TGA analysis of both empty and SPION-loaded constructs. * *p* < 0.05, ** *p* < 0.01, *** *p* < 0.001 and **** *p* < 0.0001. Figure S7: Complete statistical analysis of the in vitro cell viability-growth data based on AlamarBlue assay. AlamarBlue reduction % normalized by day 3 data for C3H10T12 mouse cells (A-B) and human bone osteoblast (HBO) (C-D) cells. Tables in B and D describe the statistical analysis labels used in the data presented in A and C. In C3H10T12 cells, significant increase of metabolic activity was observed from day 3 to days 10 and 14 (*p* < 0.0001). Significant drop was observed from day 10 to day 14 and 17 (*p* < 0.0001). The 60 µg/mL SPION-laden samples showed statistically higher viability than that in the control SPION-free constructs (*p* = 0.0282). In HBO cells, significant increase of growth was observed from day 3 and 7 to day 10 (*p* < 0.0001). It significant dropped from day 3 to 17 and from day 10 to day 14 and 17 (*p* < 0.0001). The 60 µg/mL SPION-laden samples kept statistically higher viability than the control SPION-free constructs (*p* = 0.0042). Figure S8: qPCR analysis of different cell types cultured on the HB constructs in vitro. A-C: Expression of mouse osteopontin (mOPN), osteocalcin (mOCN), and alkaline phosphatase (mALP) in C3H10T12 mouse cells after 2 weeks of in vitro culture on the HB disc constructs. D-E: Expression of human osteocalcin (hOCN) and alkaline phosphatase (hALP) in human bone osteoblast (HBO) cells after 2 weeks of in vitro culture on the HB disc constructs. * *p* < 0.05, ** *p* < 0.01, *** *p* < 0.001 and **** *p* < 0.0001. Figure S9: Micro-CT imaging of 3D bioprinted HB constructs to assess the effect of SPION incorporation on in vitro bone formation. A-C: No significant difference was observed in the total bone volume in SPION-free control group versus the 60 µg/mL SPION-laden samples after 21 days of in vitro cell culture. Figure S10: Inspection of treated rat femurs harvested at 8 and 12 weeks after surgery. A-C: Four animals were sacrificed at each time point and treated femur tissues were harvested for evaluation (left: front view, right: back view of tissue). In most groups, adequate implant engraftment and integration with the host tissue were observed, especially after 12 weeks. Figure S11: Additional scout views and µCT axial cuts of the rat femur (female and male) at 2 and 8 weeks of the in vivo study. A–D: Two female (A,B) and two male (C,D) rats, treated with scaffold, were scanned at 2 weeks after surgery. The scaffolds were well aligned within the defect site with minimal new bone formation at the bone ends. The structure of scaffold was well maintained, and no signs of mineralized callus was evident. E-H: Two female (E,F) and two male (G,H) rats, treated with scaffold, were scanned at 8 weeks post-surgery. There was evident bone formation around and within the scaffold along the whole length of the defect, with disruption of the scaffold wall, more noticeably at the plate side. Bone formation was more evident in male rats. Figure S12: Additional scout views and µCT axial cuts of the rat femur (female and male) at 12 weeks of the in vivo study, with and without scaffold. A–D: Two female (A,B) and two male (C,D) rats, treated with scaffold, were scanned at 12 weeks after surgery. There were evidences of complete bone bridging in almost all the defects, more evidently in male rats. There was fading of the scaffold structure, which was replaced by mineralized bone. E–H: Two female (E,F) and two male (G,H) rats, not treated with scaffold (control group), were also scanned at 12 weeks. The control group failed to achieve bone bridging at 12 weeks after surgery with scarce bone formation at the plate side. Video S1: 3D bioprinting of disc and cylindrical HB constructs using the 6-axis robotic arm bioprinter (BioAssemblyBot).
